# Rate of double reduction and genetic variability in yield, quality, and senescence related traits in tetraploid potato (*Solanum tuberosum L.*)

**DOI:** 10.3389/fpls.2025.1560123

**Published:** 2025-04-04

**Authors:** Muhammad Farhan Yousaf, Vipin Tomar, Hélène Romé, Merethe Bagge, Mathias Timmermann, Thinh Tuan Chu, Just Jensen

**Affiliations:** ^1^ Center for Quantitative Genetics and Genomics, Aarhus University, Aarhus, Denmark; ^2^ Research and Development, EUROPLANT Innovation, GmbH & Co. KG, Lüneburg, Germany; ^3^ DANESPO A/S, Give, Denmark

**Keywords:** potato, genetic variability, double reduction, mixed models, variance components

## Abstract

The amount of genetic variability is the foundation for genetic change in any plant breeding program, and the amount of double reduction can influence genetic gain and the amount of future genetic diversity in polyploid species. Our study investigates these factors using variance components analysis on a dataset comprising 13,131 potato breeding lines and phenotypic data from Scandinavian environments spanning 17 years (2003 to 2021). Pedigree information was used in quantitative genetic models to estimate additive genetic variance and the relative importance of additive and non-additive genetic variance. We used two models, a baseline model (M1) without effects due to specific combining ability (SCA) and M2 (including SCA due to interaction between parental genomes). Two cross-validation (CV) schemes [5-Fold and leave-one-breeding-cycle-out (LBCO)] were used to evaluate the prediction ability (PA) of each model. We estimated the rate of double reduction phenomenon (DRP) by determining the rate best fitting the data using a marginal likelihood approach. Our findings showed a wide range of variation in different traits, with very large proportion of additive genetic variance in dry matter content (DMC), but intermediate additive genetic variance for relative yield (RY), germination (GR), and withering (WNG). All traits showed modest non-additive genetic variance. Furthermore, genotype x environment interaction played a significant role in trait variability but is still much smaller than the additive genetic variance. After using different DRP rates, we found that a model with a 0.05 DRP rate provided the best fit to the data. Heritability estimates indicated a strong genetic basis for DMC, while other traits showed more moderate heritability, which shows contributions from both additive and interaction factors. Model comparison by 5-Fold CV and LBCO and the log likelihood ratio test (LRT) highlighted the importance of considering SCA when capturing trait variability. In 5-Fold CV, PA ranged from 0.296 to 0.812 in M1 and 0.300 to 0.813 in M2. Under LBCO CV, PA ranged from 0.180 to 0.726 in M1 and 0.180 to 0.728 in M2. However, an increase in PA in Model 2, which incorporates SCA, compared to Model 1, can be attributed to the inclusion of SCA effects. Furthermore, the LRT results indicated a highly significant difference between the models. CV and LRT suggest the need for genetic models that account for both additive and SCA effects. Our analysis also showed that genotype x environment interactions should be accounted for in order to maximize the accuracy of predicted breeding values of tetraploid potato clones. The rate of double reductions was small and insignificant.

## 1 Introduction

Potato (*Solanum tuberosum* L.) is one of the most significant vegetal crops across the globe and is the fourth most important food crop after rice, wheat, and maize (FAOSTAT, 2022). It is a member of the asterid group of eudicot plants, which denotes approx. 25% of all flowering plants. Potato is adapted to wide ecological and geographical ranges and is foremost in mass-producing stolons among the global food crops (Hijmans, 2001). Potato breeders are primarily interested in contributing to genetic gain to deliver future food security at the global level, as well as providing economic benefits to potato breeding companies and potato growers.

The potato is extremely heterozygous, and therefore, many traits in potatoes can be affected by both additive and non-additive genetic effects. In general, additive genetic effects contribute to General Combining Ability (GCA) while Specific Combining Ability (SCA) is mainly a function of non-additive genetic effects (Falconer, 1996). GCA effects are primarily used for breeding because they include additive genetic effects that are inherited by the next generations. The SCA can be used to select commercial varieties/crosses but is not important for long-term genetic gain in the breeding population because non-additive effects are lost in future generations due to recombination. Knowledge of the genetic variance components (VCs) contributing to GCA and SCA is required to be able to design optimal breeding programs, estimate expected genetic response from a genetic selection program, and is a required parameter in mixed models for prediction of genetic effects. The relative importance of GCA and SCA depends on the specific traits under analysis, the population studied, and the environmental conditions where the population is grown. Brown and Caligari (1989) found that GCA effects were significant for three traits tested (total tuber weight, number of tubers, and breeder’s preference), whereas SCA was only significant for mean tuber weight. Furthermore, the interaction of test sites with GCA was significant for total tuber weight and very significant for average tuber weight. This implies that the performance of crossings may be predicted based on the phenotype of the parents, with GCA playing an important role in determining the superior crosses/matings.

The findings suggest that the primary importance of GCA is in identifying better parents and forecasting progeny performance in potato breeding programmes. Similarly, Maris (Maris, 1990) performed a study to compare the performance of diploid (2x) and tetraploid (4x) potatoes, focusing particularly on tuber yield, and concluded that ploidy (2x and 4x) influences phenotypic variation among potato families. While GCA was evident in the significant differences observed among the families, the discovery of strong SCA effects and genotype by environment interactions emphasises the necessity of taking non-additive genetic factors into account in potato breeding programmes. Ruiz de Galarreta et al. (2006) performed a partial diallel cross using 14 potato cultivars chosen for fertility. They assessed the progeny throughout numerous generations for yield, tuber number, and average tuber weight. Significant diversity was found across all traits and generations due to both general and specific combining ability, with SCA having the largest influence.

Potato cultivars exhibit significant diversity, as evidenced by extensive allelic diversity, altered coding and transcript sequences, preferential allele expression, and structural variation observed across six cultivars in the study conducted by Hoopes et al. (2022). Understanding this genetic variability is crucial for investigating the genetic basis of yield, quality, and senescence related traits in tetraploid potato. However, environmental variability, for example, seasonal variation, site effect, and their interactions, greatly affects production (Aina et al., 2021). Unpredictable yield losses of potato crops across years and sites are mainly due to numerous biotic and abiotic effects, along with the absence of widely adapted and early maturing varieties (Acevedo et al., 2020; Tiwari et al., 2022), underscoring the need for improved potato varieties that can adapt to diverse environmental conditions. Plant breeders utilize information on the genetic components of traits and the level of genetic variability amongst locally grown varieties to foresee the requirement for the development of new improved varieties in breeding (Uzun et al., 2013).

**Figure 1:**
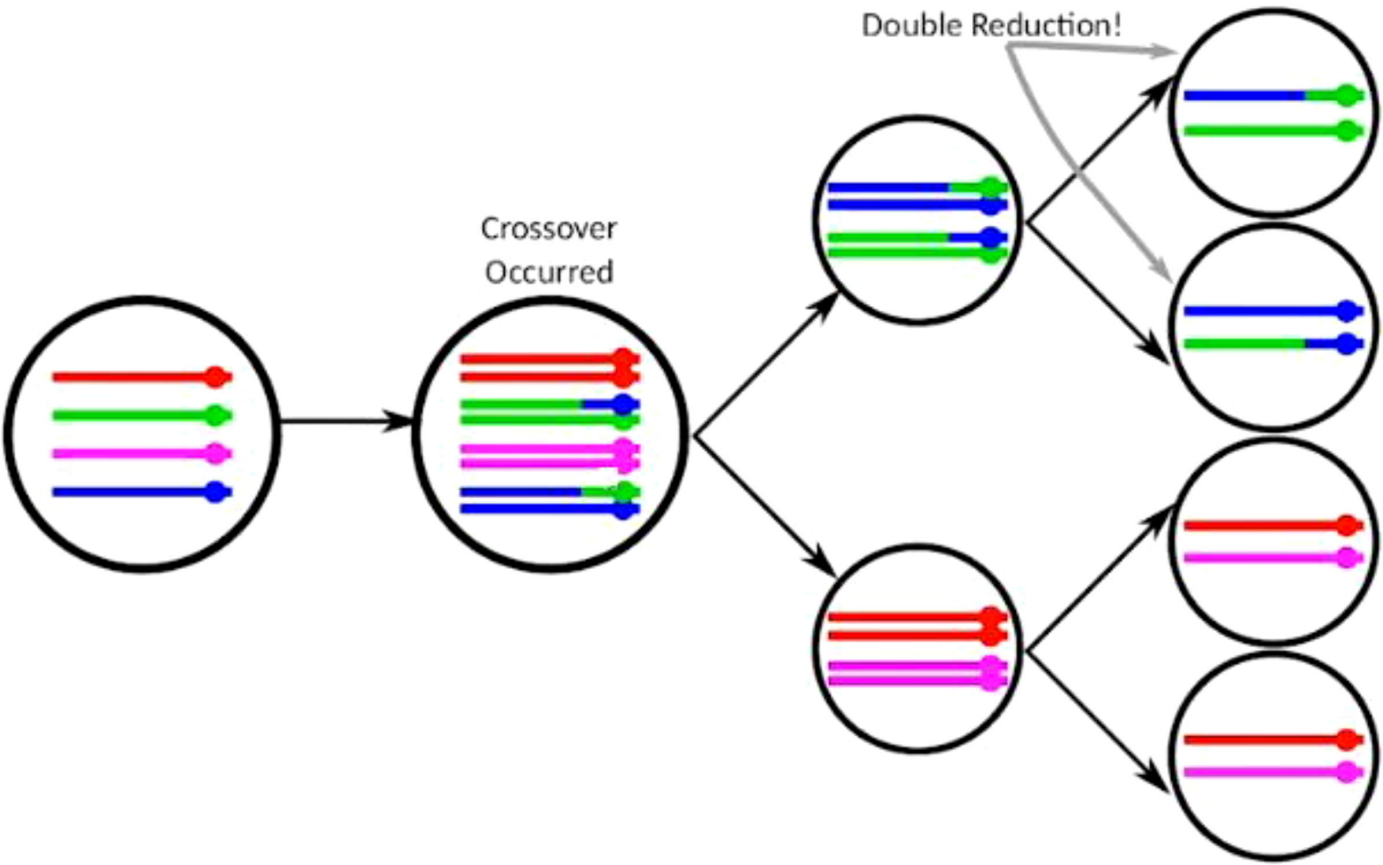
Double reduction phenomenon in tetraploid crops. Modified from the video “Double Reduction Estimation and Equilibrium Tests in Natural Autopolyploid Populations” presented by David Gerard dated 24.11.2021.

Polyploidy refers to the condition where an organism has more than two complete sets of chromosomes. It can occur naturally and is common in many plant species. Polyploids can be categorized as either autopolyploids, where the extra sets of chromosomes come from the same species, or allopolyploids, where the additional sets come from different species (Otto and Whitton, 2000; Udall and Wendel, 2006; Soltis et al., 2004). Potato is an autopolyploid with a chromosome number of 2n = 4x = 48 and a genome size of 844 megabase pairs (Mbp). Therefore, homologous chromosomes can be paired to create multivalents in the meiosis phase. Then, due to crossing-over and chiasma formation, alleles at target loci from sister chromatids can be delivered to the same gamete. This process is called the double reduction phenomenon (DRP) (Milbourne et al., 2008; Bradshaw, 2017) (**Figure 1**). In conjunction with random bivalent pairing during prophase I of meiosis, the coupling behavior of potato is reasonably well explained (Sawaminathan and Howard, 1953; Milbourne et al., 2008; Bourke et al., 2015) compared to other autotetraploid species such as rose, rice, and alfalfa (Cao et al., 2004; Bourke et al., 2017; Li et al., 2020; Liu et al., 2023). The DRP evaluation is generally performed from two known genotypes/parents by developing and analyzing a population of crosses (Haynes and Douches, 1993; Wu et al., 2001). DRP may play an important role in genetic drift (Moody et al., 1993) and in gametophytic selection (Butruille and Boiteux, 2000). Veteläinen et al. (2005) came to the conclusion that Nordic countries have a great diversity of potato landraces, which vary in many traits and have a high potential to improve the potato crop. The variability present in the crop germplasm is vital for the breeding of improved varieties.

This study aimed to estimate population parameters in term of variance components for traits related to yield, quality, and senescence, as well as the amount of genotype by environment interaction for varieties grown under Danish conditions. It also aimed to determine the level of DRP in the population studied. The estimated variance components will be used to derive the importance of GCA and SCA and validate the models developed using cross validation strategies and standard likelihood ratio tests.

## 2 Materials and methods

### 2.1 Plant material

Breeding lines from a large-scale potato breeding program tested in Denmark (2003 to 2021) were used for the present study. These lines or varieties were developed and maintained by Danespo A/S as part of a long-term breeding program. The lines used in the present study were developed for different market segments, including table, starch, and crisp potatoes. The breeding program included phenotyping from preliminary to elite trials, and these lines were chosen to represent all the trials (preliminary to elite) at a given period. New clones from each market segment consisted of clones in the second to fifth year after the initial cross and were tested in trials specific to their respective market segments. The distribution of data on years and trials was very uneven, as some trials were missing in some years, good genotypes were assessed in numerous trials and replicates, and poor clones were tested in only a single trial, which is usual for a breeding program since not all genotypes are evaluated in all trials. For example, 76%, 14%, and 10% of the genotypes were tested in a single trial, two trials, and >3 trials, respectively.

### 2.2 Phenotypic data collection

A total of 13,131 lines were scored for dry matter content (DMC), relative yield (RY), germination rate (GR), and withering trait (WNG). DMC is the percentage of a sample’s mass that remains after all water has been removed. It is based on weighing above and underwater and calculated using the formula shown in **Supplementary Table S1**. RY is a measure of yield relative to all tests of the same market segment within a given test year and site. GR is the percentage of planted tubers that successfully germinate within a given period. WNG refers to the degree to which a plant exhibits signs of withering. The GR and WNG traits were evaluated three times. The germination assessment began approximately 2 weeks after planting, depending on the weather. The withering evaluation started when the first clones showed signs of dying off and was also conducted three times. A detailed explanation of the traits analyzed is provided in **Supplementary Table S1**.

### 2.3 Pedigree information

The total size of the pedigree available included 59,289 individuals, which was used to trace the pedigree for the lines under study. We utilized the DMUTrace software (Madsen et al., 2014) to trace the pedigree of the phenotyped lines and assess the completeness of the pedigree. Completeness was expressed as the number of known generations of ancestors for each individual. In total, 897 lines had missing information on one or both parents. If both parents of an individual were known, the completeness of this individual was 1. If all grandparents of the individual were also known, the completeness of the individual was 2. Only 6% of lines had unknown parents, and the remaining 94% of the lines had known parents/generations, with 24% having both parent information and 70% having information about both parents and grandparents. The maximum number of known generations for an individual was 4.90. The average completeness of the pedigree was 2.39. Relationships between the lines or varieties were determined using pedigree information. The pedigree file corresponding to all clones with data was arranged chronologically using DMUtrace (Madsen et al., 2014) software and was further analyzed by the AGHmatrix R package (Amadeu et al., 2016) to calculate the additive relationship matrix (**A**) accounting for the tetraploid nature of potato and the possibility of double reduction. Different pedigree relationship matrices were built assuming different DRP frequencies (0.05 to 0.9). The completeness of the pedigree was further subdivided by year and market segment and presented in the **Supplementary Material** (**Supplementary Table S2**).

### 2.4 Statistical methods

Quality control protocols were used to prevent errors and ensure the precision and dependability of our results. It was done using R and SPSS software. Discrepancies such as typographical errors and outliers were observed by plotting box plots and corrected, and assumptions of normal distributions were checked using histograms. All the data generated were further analyzed using DMU statistical software version 6 (Madsen et al., 2014).

In this study, two mixed models were used to estimate the rate of double reduction and the amount of genetic variability of studied traits. First, a baseline mixed model (M1) with no SCA was used as a starting point for building the other model. Before using this baseline model as a starting point, many other factors were tested to analyze the relationship between the traits studied and genetic factors. Only significant factors (P<0.05) were included in the model. These models showed promise in capturing intrinsic variability while noting that these models had not yet included SCA. After preliminary experimentation, M1 was chosen based on the likelihood ratio test (LRT) and the significance of variance components. Second, the baseline model was extended by including an SCA effect (M2), estimating the non-additive genetic variation contributed by the interaction between specific combinations of individuals as parents. The AI-REML (Average Information Restriction Estimation Maximum Likelihood) algorithm in DMU software (Madsen et al., 2014) was used for variance components estimation using bivariate models including for DMC and RY, and for GR and WNG. The models used were:


(1) y = Xb  +Z₁a + Z₂g+ Z₃i +ϵ

From the baseline model (1), the SCA random effect was added:


(2) y = Xb + Z₁a + Z₂g+ Z₃i + Z₄s +ϵ

where **y** is a vector of phenotypes and **b** is the vector of fixed effects, which included effects due to trial/market segment, year, and their interaction between trial and year on phenotypic response variable. a, g, i, and s are random effects that come from independent, normal distributions, where **a** is a vector of additive genetic effect with **a ∼ N(0, A** $⊗\left[\begin{array}{cc} \sigma_{a1}^{2} & \sigma_{a1a2}\\ \sigma_{a1a2} & \sigma_{a2}^{2} \end{array}\right]$ **), A** is the pedigree relationship matrix, $\sigma_{a1}^{2}$ , $\sigma_{a2}^{2}$ and $\sigma_{a1a2}$ are additive genetic variances of trait 1 and 2, and the covariance, respectively. ***g*** is a vector of line effect with **g ∼N(0,I ⊗** $\left[\begin{array}{cc} \sigma_{g1}^{2} & \sigma_{g1g2}\\ \sigma_{g1g2} & \sigma_{g2}^{2} \end{array}\right]$ **),** where $\sigma_{g1}^{2}$ , $\sigma_{g2}^{2}$ and $\sigma_{g1g2}$ are line variances of traits 1 and 2 and covariances respectively, and **I** is an identity matrix. ***i*** is a vector of interaction effect of genotype × trial × year interaction (G×T×Y) with **i∼N(0,I** $\;⊗\left[\begin{array}{cc} \sigma_{i1}^{2} & \sigma_{i1i2}\\ \sigma_{i1i2} & \sigma_{i2}^{2} \end{array}\right]$ **),** follow a bivariate normal distribution with a mean of 0, where $\sigma_{i1}^{2}$ , $\sigma_{i2}^{2}$ and $\sigma_{i1i2}$ are the interaction (co)variances of traits 1 and 2. ***s*** is a vector for SCA with **s∼N(0,I** $\;⊗\left[\begin{array}{cc} \sigma_{s1}^{2} & \sigma_{s1s2}\\ \sigma_{s1s2} & \sigma_{s2}^{2} \end{array}\right]$ **),** follows a bivariate normal distribution (N) with a mean of 0 where $\sigma_{s1}^{2}$ , $\sigma_{s2}^{2}$ and $\sigma_{s1s2}$ are the SCA variances of traits 1 and 2, and covariances respectively. ***ϵ*** represents the residual effect or error term with $\in \sim N(0,S\;⊗\left[\begin{array}{cc} \sigma_{e1}^{2} & \sigma_{e1e2}\\ \sigma_{e1e2} & \sigma_{e2}^{2} \end{array}\right]$ **),** where $\sigma_{e1}^{2}$ , $\sigma_{e2}^{2}$ and $\sigma_{e1e2}$ are the residual variances of traits 1 and 2 and covariances respectively**. X** and **Z_j_** are design matrices for fixed and random effects, respectively. Fixed effects included trial, year, and their interaction, while the random effects capture the random variation due to the grouping structure of the data (additive genetic effect, remaining non-additive genetic effects, genotype×trial×year interaction effects, and SCA as the interaction between parent 1 and parent 2 of individual lines). SCA effects were added in the second model, where SCA refers to the non-additive genetic effects that arise due to the interaction between genes originating from different parents (parent 1 and parent 2). The mixed model methodology permits the estimation of ***b*** by the generalized least squares and the prediction of the random effect by the best linear unbiased predictions (BLUP) procedure. The models were run using different assumptions on the rate of double reductions and the log-likelihood was then used to compute a marginal likelihood profile. The maximum of this likelihood was used to estimate the rate of DR in the population. All rates of DRP below 0.05 yielded the same log-likelihood, and we, therefore, chose only to report results from a rate of DRP of 0.05 and onwards.

### 2.5 Heritabilities

Broad-sense (H^2^) and narrow-sense (*h^2^*) heritability at the plot level were computed using the estimated variance components as follows.


(3) $$\hat{{\text{H}}^{2}}=\frac{\left(\text{d}\left(\text{A}\right)\hat{\sigma_{\text{a}}^{2}}\right)+\hat{{\text{σ}}_{g}^{2}}+\;\hat{{\text{σ}}_{s}^{2}}}{\left(\text{d}\left(\text{A}\right)\hat{\sigma_{\text{a}}^{2}}\right)+\hat{{\text{σ}}_{g}^{2}}+\hat{{\text{σ}}_{i}^{2}}+\hat{{\text{σ}}_{s}^{2}}+\hat{{\text{σ}}_{e}^{2}}}$$

(4) $$\hat{h^{2}}=\frac{\left(\text{d}\left(\text{A}\right)\hat{\sigma_{\text{a}}^{2}}\right)}{\left(\text{d}\left(\text{A}\right)\hat{\sigma_{\text{a}}^{2}}\right)+\hat{{\text{σ}}_{g}^{2}}+\hat{{\text{σ}}_{i}^{2}}+\hat{{\text{σ}}_{s}^{2}}+\hat{{\text{σ}}_{e}^{2}}}$$

The above formula corresponds to M2. For the M1 model, H^2^ and *h* ^2^ were calculated without SCA. d(A) is the average diagonal of the additive genetic relationship matrix. The heritability presented refers to heritability at the plot level, i.e., the heritability of measurement on a single plot. The heritability of a clone means assuming a different level of replication that can easily be computed from the information provided.

### 2.6 Assumption on DRP affecting the relationship matrix A

**Figure 2:**
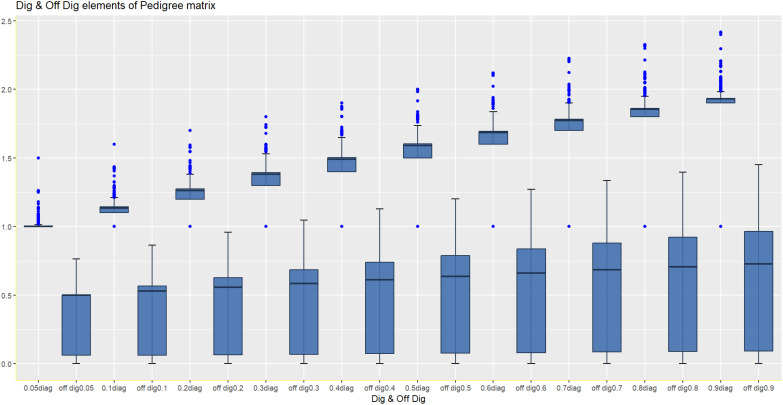
Effect of different assumptions of the DRP on the relationship matrix (diagonal and off-diagonal elements of A).

The double reduction phenomenon is genetic and occurs during meiosis. It can impact the distribution of alleles and expected additive genetic relationships among individuals. In potato breeding, the DRP increases the complexity of the additive genetic relationship matrix **A**. Different assumptions of the occurrence of the DRP significantly impact the diagonal elements of **A** through changes in genetic diversity and genetic structure (**Figure 2**). The off-diagonal elements represent the pairwise relatedness between individuals, and the DRP can have notable effects on the expected relationships between clones, especially when shared ancestors have undergone the DRP. **Figure 2** depicts how the double reduction phenomenon changes the additive relationship matrix **A** by affecting both the relatedness of individuals to themselves (inbreeding) and the relatedness of individuals to other individuals (off-diagonal elements), i.e., individual lines become more related. The strategy used for estimating the rate of the DRP is in line with methodologies used in numerous studies, including the development of accurate models for leaf area prediction in loquat cultivars, as evidenced by Teobaldelli et al. (2019) in their study on *Eriobotrya japonica*.

### 2.7 Model validation by assessing prediction accuracy using cross-validation

To validate the models used for the prediction of genetic effects, the present study used two cross-validation (CV) schemes: *5*-Fold (Burgueño et al., 2012) and leave-one-breeding-cycle-out (LBCO) for each prediction model. In brief, for 5-Fold CV, the clones were divided into five random non-overlapping subsets. Then, one group served as the testing set, while the remaining groups served as the training set [breeding values (BV) were predicted for each clone in the testing set]. This procedure was repeated until breeding values from all 5 subsets were predicted (**Supplementary Figure S1**). A 5-fold CV provides a reliable model comparison, helps in model selection, and aids in understanding the bias-variance trade-off. For LBCO CV, the lines were organized based on their crossing year, and for each crossing year, the lines were checked for the presence of their parents in the rest of the data. If a parent was present anywhere (either as parent 1 or parent 2), they were included in that crossing year and were considered a breeding cycle. In this way, all full and half siblings were masked for each line within one breeding cycle. The masked breeding cycle/year was predicted using the remaining breeding cycles (**Supplementary Figure S2**). This procedure was repeated for each breeding cycle (number of breeding cycles = 28) until all breeding cycles were predicted. The LBCO emulates the prediction problem in which a new generation (newly developed lines) is predicted based on parental and historical data and is, therefore, very relevant in a breeding context.

**Figure 3:**
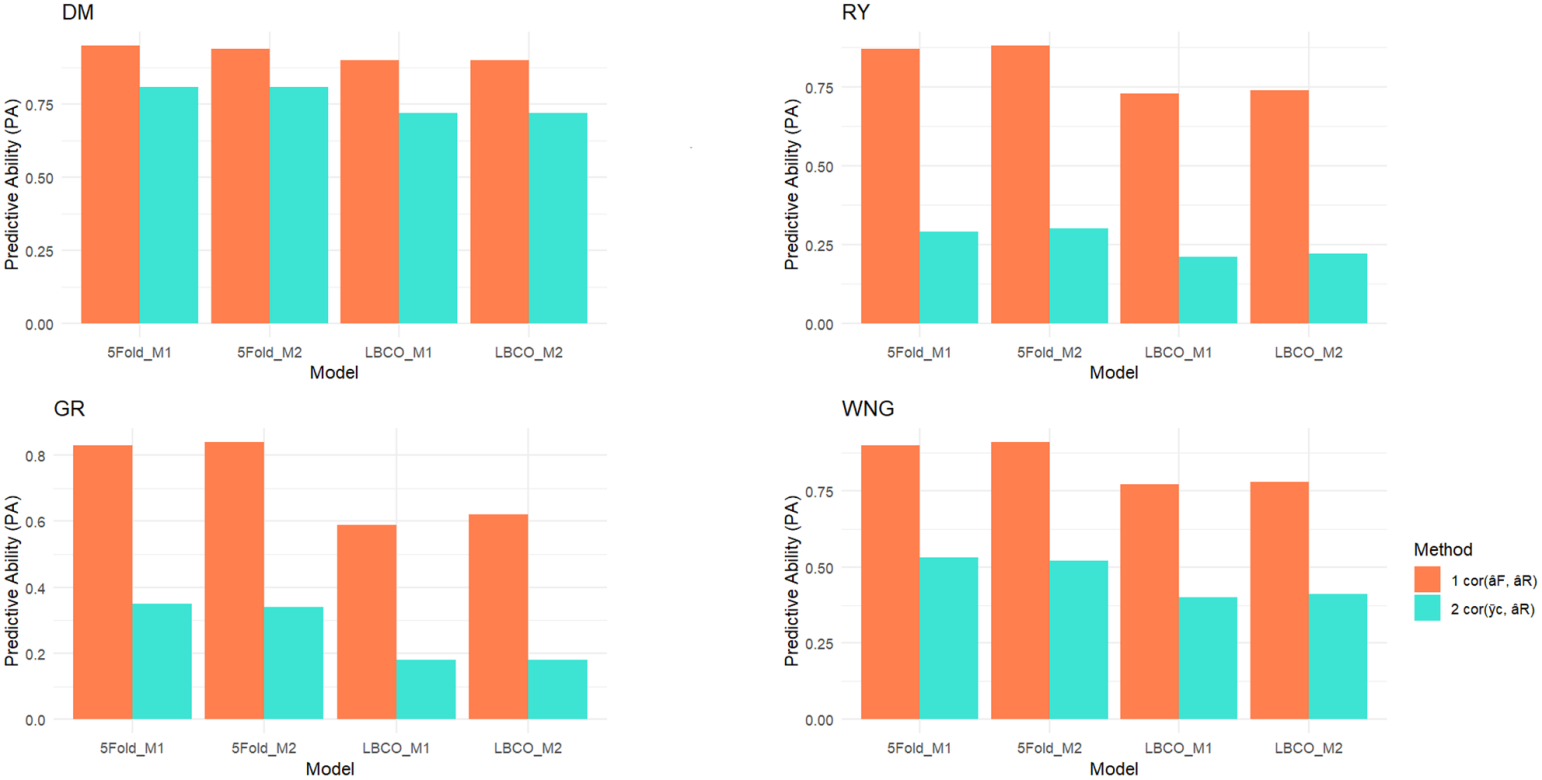
Predictive abilities in 5-Fold and leave-breeding-cycle-out (LBCO) cross-validations and a comparison of model 1 and model 2 for potato dry matter content (DMC), relative yield (RY), germination (GR), and withering features (WNG) traits. Furthermore, the theoretical maximum of $cor\left(\bar{y_{c}},\hat{a_{R}}\right)$ , which was calculated as $\sqrt{\hat{h_{\bar{y_{c}}}^{2}}}$ , providing a reference for the performance of our prediction models (**Supplementary Table S5**). The complete formula for the theoretical maximum of $cor\left(\bar{y_{c}},\hat{a_{R}}\right)$ is provided in the **Supplementary Material** as Equation 1.

The predictive ability (PA) of the models was evaluated using the Pearson correlation between the average value of lines after correcting for fixed effects and the vector of predicted breeding values, $cor\left(\bar{y_{c}},\hat{a_{R}}\right)$ . The fixed effects were estimated for each model using the full dataset to obtain as accurate estimates as possible. The phenotypes were corrected for the fixed effects by subtracting the estimated fixed effects from each observation $\hat{y_{c}}=\left(y-X\hat{\beta }\right)$ and then average corrected phenotypes ( ${\bar{y}}_{c}$ ) were computed for each clone or line. The correlation between ${\bar{y}}_{c}$ and predicted breeding values can at most be the square root of heritability of ${\bar{y}}_{c}$ (**Figure 3**; **Supplementary Table S5**) (Crossa et al., 2010; Kristensen et al., 2019). The relative change in prediction accuracy of breeding values (RC) when going from the reduced to the full dataset was calculated as the correlation between full model prediction ( $\hat{a_{F}}$ ), which is the breeding values estimated with complete phenotypic information for all the lines, and breeding values when phenotypic information was partially masked ( $\hat{a_{R}}$ ), I.e. $cor\left(\hat{a_{F}},\hat{a_{R}}\right)$ (Legarra and Reverter, 2018).

## 3 Results

This study used 13,131 breeding lines for all the studied traits under 12 different trial types maintained by the DANESPO potato breeding program from 2003 to 2021. Simple descriptive statistics are summarized in **Table 1**. In general, the population displayed a wide range of variation, with all the traits exhibiting a wide range between the minimum and maximum phenotypic values. For example, DMC ranged from 11 to 34 with an average of 23.5. Similarly, RY ranged from 23 to 251 having an average of 96.3. WNG ranged from 2 to 9 with an average of 5.9, while GR ranged from 1 to 9 with an average of 4.5.

**Table 1:** Descriptive statistics of the studied traits.

Trait	N-lines	N-obs	Average	SD	Min	Max
DMC	13131	24284	23.5	4.1	11	34
RY	13131	23878	96.3	19.5	23	251
WNG	13131	24669	5.9	1.2	2	9
GR	13131	27729	4.5	1.3	1	9

DMC, dry matter content; RY, relative yield; WNG, withering; GR, germination; N-obs, number of observations; N-lines, number of lines/individuals; SD, standard deviation.

### 3.1 Estimates of DRP based on marginal log-likelihoods

Different expected frequencies of the DRP, i.e., 0.05, 0.1, 0.2, 0.3, 0.4, 0.5, 0.6, 0.7, 0.8, and 0.9, were considered to estimate the amount of DRP in the population. The marginal log-likelihood values were obtained for each assumed frequency of the DRP using both M1 and M2. The models that assumed that the rate of DRP was smaller than 0.05 yielded the same results as those that used a rate of 0.05, and these results, therefore, were not presented.

We found that with an expected DRP of 0.05, the -2*loglikelihood value (-2LogL) was minimum (likelihood maximized) for all traits investigated. After estimating the rate of DRP, which was consistent across all the traits analyzed, the focus moved to estimating the genetic parameters (variance parameters and breeding values) while assuming a DRP rate of 0.05.

### 3.2 Variance components of the studied traits under the optimal model (0.05 DRP)

**Figure 4:**
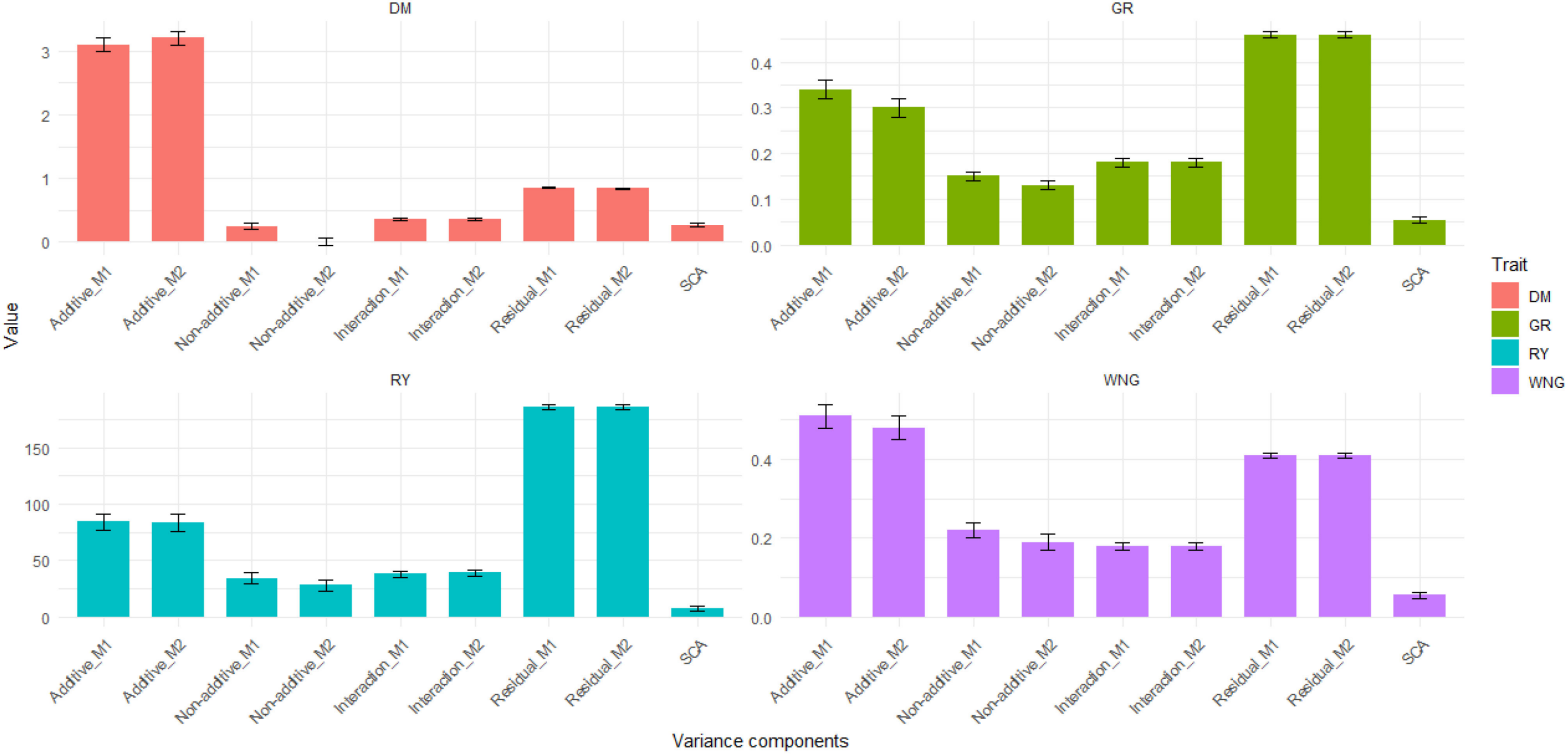
Bar graph showing variance components obtained by models M1 and M2 for dry matter content (DMC), relative yield (RY), germination rate (GR), and withering (WNG).

VCs were estimated by two models, i.e., the M1 model without SCA and the M2 model with the SCA effect included. The results, shown in **Figure 4**, revealed that the additive genetic variance ( $\sigma_{a}^{2}$ ) and residual variance ( $\sigma_{e}^{2}$ ) accounted for the largest proportion of variance compared to the other variance components. A detailed account of the estimates can be found in **Supplementary Table S3**.

#### 3.2.1 Dry matter content

The estimated additive genetic variance ( $\sigma_{a}^{2}$ ) was 3.1 ± 0.1 in M1 and 3.2 ± 0.1 in M2, which was high compared to other VCs in the model and showed that the additive genetic component has a substantial influence on the variation observed in the trait. Furthermore, the non-additive genetic effect showed less influence than the interaction and residual variances. Notably, the interaction effect was consistent across both models, implying uniform levels of genotype-environment interaction in DMC in both models (**Figure 4**). Additionally, additive genetic variance explained 67.9% of the total phenotypic variance ( $\sigma_{p}^{2}$ ) while the residual variance ( $\sigma_{e}^{2}$ ) contributed 18.6%, with other variance components collectively explaining the remaining 5.4% to 7.8% in the baseline model (M1). In the SCA model (M2), a similar trend was observed where additive genetic variance ( $\sigma_{a}^{2}$ ) of 3.2 at 0.05 DRP explained approximately 68.3% and residual variance ( $\sigma_{e}^{2}$ ) 17.1% of the total phenotypic variance. Notably, the SCA accounts for variance that was previously attributed to non-additive variance components in the baseline model (M1), explaining about 5.7%. The SCA effects are due to non-additive interactions between alleles inherited from different parents.

#### 3.2.2 Relative yield

The genetic variance ( $\sigma_{a}^{2}$ ) was 84.3 ± 7.4 which explained 24.6% of the total phenotypic variance among plots ( $\sigma_{p}^{2}$ ), suggesting a stronger additive genetic influence on this observable trait as compared to other VCs (**Figure 4**). Furthermore, the non-additive genetic variance ( $\sigma_{na}^{2}$ ) were lower compared to the other VCs and explained 10.05%, while the residual variance ( $\sigma_{e}^{2}$ ) explained 54.1% of the total phenotypic variance ( $\sigma_{p}^{2}$ ) in M1. In M2, when SCA was included in the model, there was a reduction in both additive genetic variance (from 84.3 ± 7.4 to 83.3 ± 7.8) and non-additive genetic variance (from 34.5 ± 5.1 to 28.0 ± 5.4), indicating that the SCA component accounts for some of the genetic variance previously attributed to additive and non-additive genetic influences and it was 7.5 ± 2.0 (**Figure 4**). Surprisingly, the interaction and residual variances exhibited a modest increase. The observed increase with the inclusion of SCA suggests that the model better accounts for additional sources of variation caused by interactions between parental genotypes. These findings highlight the importance of considering SCA in genetic models, as it contributes to a better understanding of the genetic architecture underlying the observed trait. Finally, the ( $\sigma_{a}^{2}$ ) explained approximately 24.2% and residual variance ( $\sigma_{e}^{2}$ ) 54.06% of the total phenotypic variance.

#### 3.2.3 Germination rate

VCs revealed that both additive genetic variance ( $\sigma_{a}^{2}$ ) (0.34 ± 0.02 in M1 and 0.30 ± 0.02 in M2) and residual variance ( $\sigma_{e}^{2}$ ) (0.46 ± 0.006 in both models) were the dominant sources of variation in GR, while non-additive genetic effects ( $\sigma_{na}^{2}$ ) and interaction effects ( $\sigma_{i}^{2}$ ) contributed to a lesser extent. Interestingly, the interaction effect was found to be more influential than the non-additive genetic effect (**Figure 4**). Additive variance explained approximately 30.08% of the total phenotypic variance ( $\sigma_{p}^{2}$ ) while the residual variance ( $\sigma_{e}^{2}$ ) contributed 40.70% and other variance components collectively explained the remaining 13.2%. to 15.9%. In the SCA model (M2), a similar trend was observed where additive genetic variance ( $\sigma_{a}^{2}$ ) explained approximately 26.6% and residual variance ( $\sigma_{e}^{2}$ ) 40.90% of the total phenotypic variance ( $\sigma_{p}^{2}$ ) (**Supplementary Table S3**). The SCA captures 4.80% of the total phenotypic variance, which was attributed to non-additive variance components in the baseline model (M1).

#### 3.2.4 Withering

Like for the other traits analysed, the estimated additive genetic variance ( $\sigma_{a}^{2}$ ) was high (0.51 ± 0.03 in M1 and 0.48 ± 0.03 in M2) compared to the other VCs (**Figure 4**) and showed that the additive genetic component ( $\sigma_{a}^{2}$ ) has substantial influence on the variation observed in the trait, ( $\sigma_{a}^{2}$ ) explaining 38.63% of the total phenotypic variance ( $\sigma_{p}^{2}$ ), while the residual variance ( $\sigma_{e}^{2}$ ) contributed 31.06%, and other variance components (non-additive genetic effects, SCA, and G*E) collectively explained the remaining 13.6% to 16.6%. In the SCA model (M2), a similar trend was observed where the additive genetic variance ( $\sigma_{a}^{2}$ ) explained 36.47% and residual variance ( $\sigma_{e}^{2}$ ) 31.15% of the total phenotypic variance ( $\sigma_{p}^{2}$ ). The reduction of genetic variance components after the addition of the SCA component showed that in M1, it was attributed to additive and non-additive genetic variances (**Figure 4**). These findings emphasise the necessity of considering SCA in genetic models, which leads to a more comprehensive understanding of the genetic architecture underlying the observed characteristic.

### 3.3 Estimation of heritability

**Figure 5:**
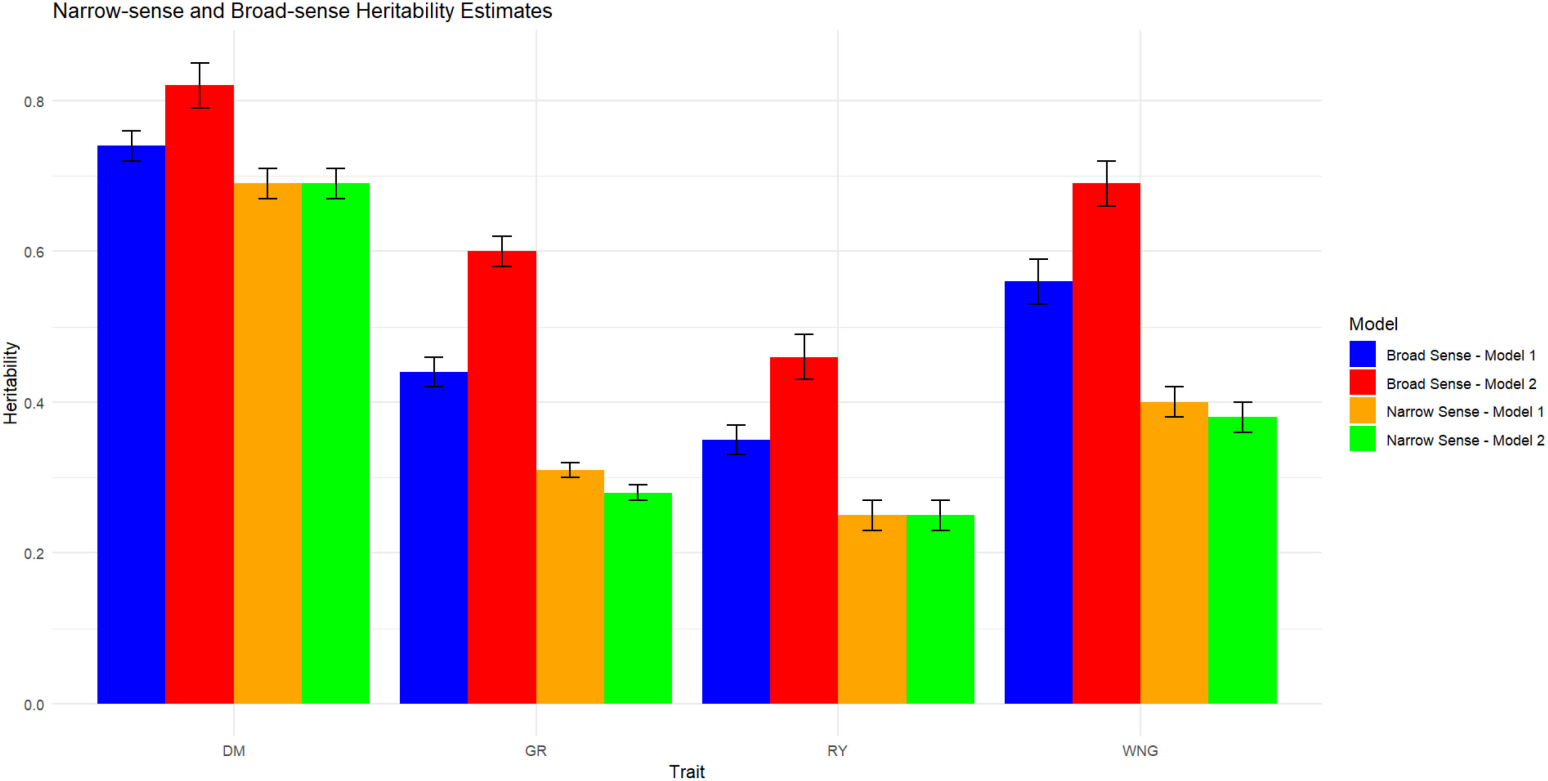
Estimates of the narrow- and broad-sense estimates of heritability at 0.05 DRP in both models. DMC, dry matter content; GR, germination; RY, relative yield; WNG, Withering.

Heritability below 30% is considered low, while medium is between 30% and 60%, and high is above 60%. In the present study, the *h^2^* was estimated in both M1 and M2 models and expressed as the heritability of a single plot measure. For DMC, the estimates were 0.69 in M1 and 0.70 in M2 model and H^2^ was 0.75 in M1 and 0.82 in M2 model (**Figure 5**). This shows that genetic factors play major roles in determining DMC. Further details can be found in **Supplementary Table S3**.

The *h^2^* estimate for RY was 0.26 in M1 and 0.25 in M2 model suggesting a moderate impact of additive genetic effects on the trait (**Supplementary Table S3**). However, H^2^ increased from 0.36 in M1 to 0.46 in M2 suggesting that genetic effects, both additive and non-additive (e.g., dominance and epistasis) captured by SCA, account for a significant fraction of the phenotypic diversity in RY.

In M1, the narrow-sense heritability of the GR trait was estimated to be 0.31 while in the M2 model it was 0.28. In contrast, the broad-sense heritability values in the M1 model were estimated at 0.44, whereas in the M2 model it was 0.60. The narrow-sense heritability of the WNG trait was 0.40 in the M1 model and 0.38 in the M2 model. Broad-sense heritability estimates was found to be 0.56 in M1 and 0.69 in M2. Interestingly, when the SCA effect was included in the model, we observed a modest increase in broad sense heritability estimates across all the traits compared to the baseline model (model without SCA). This could be due to the specific combining ability component, which captures non-additive genetic effects due to interactions between parental genomes. Narrow sense heritability estimate was observed to be low except for DM in M2 (with SCA) which means including specific combining ability in a model redistributes part of the variation in the trait to non-additive genetic effects.

### 3.4 Cross-validation

The models were validated using 5-Fold and LBCO cross-validation. The validation results are presented as PA and RC in the accuracy of breeding values when going from predicting breeding values based on the reduced dataset to predicting based on the full data, i.e., the change in accuracy of predicted breeding values when the masked data is added to the model. The results are shown in **Figure 3**, and more details can be found in **Supplementary Table S4**.

The PA for the two models were similar in the 5-fold CV. The accuracy of the predicted breeding values for the lines with their phenotypes masked ranged from 0.296 for RY to 0.812 for DM. The RC ranged from 0.839 in WNG to 0.950 in DM in M1. In comparison, the accuracy was 0.300 in RY and 0.813 in DM, and the RC ranged from 0.849 in GR to 0.949 in DM trait in M2 model. Interestingly, when the SCA model (M2) was compared to the model without SCA (M1), the RC in both models were practically identical for DMC, but a considerable improvement was noticed in RY, GR, and WNG traits in M2. The PA was slightly improved in DMC and RY.

The LBCO CV evaluates the accuracy that can be obtained when making decisions for future years not yet included in the data. The prediction accuracy ratio is improved in M2 compared to M1. The PA of values ranged from 0.180 in GR to 0.726 in DM, and RC was 0.599 in GR and 0.904 in DM in the M1 model as compared to an average accuracy of 0.180 in GR and 0.728 in DM and the accuracy ratio was between 0.627 in GR and 0.906 in DM trait in the M2 model. In LBCO, it was observed that PA was improved in the DM, RY, and WNG traits in M2 (with SCA), but for RY, it was the same as M1.

### 3.5 Model comparison using standard likelihood ratio test

We used the LRT to compare the fit of the two mixed models in explaining the observed variance in the traits studied. The models included the M1 model (without the SCA effect) and the M2 model (with the SCA effect as a random effect).

The LRT results indicated a statistically significant difference in model fit between the two tested bivariate mixed models (M1 and M2) for DMC and RY, yielding a P< 1.57E-22 at 0.05 DRP with three degrees of freedom. The analysis was conducted in a bivariate model, where the combined effect of both traits was tested. Therefore, the p-value reflects the shared model fit improvement for both traits simultaneously. Similarly, for the traits GR and WNG, the LRT test resulted in P< 8.96E-35, also with three degrees of freedom (df = 3). The obtained p-values from the LRT strongly suggest a highly significant difference in model fit between the mixed models in favor of M2.

M2 showed a significantly better fit to the observed data compared to the alternative model M1, suggesting that it included components or effects within the SCA model that contributed to the explanation of observed variability. This implies that the SCA effect included in the model better captures non-additive genetic effects due to interaction between alleles inherited from the specific combination of two parents used in a given mating.

## 4 Discussion

The amount of additive genetic variability is directly related to the potential genetic gain in any breeding program. We used 13,131 potato lines (clones), maintained by DANESPO A/S, to estimate the rate of double reductions and population parameters for dry matter content, relative yield, germination, and withering. Pedigree information was used to calculate the numerator relationship matrix **A** for the estimation of additive genetic variance and estimation of the amount of DRP in the population. We defined models (M1 and M2) to uncover the relative importance of additive genetic, non-additive, and other variance components, especially variance due to specific combining ability and genotype by environment interaction. In the M2 model, a parent-by-parent interaction effect was included to account for the SCA effect due to the interaction between parental genomes. Furthermore, the PA for both models was investigated using two CV schemes: (i) 5-Fold and (ii) LBCO. Generally, the population used in the study displayed a wide range of variation, with all traits exhibiting a wide range between the minimum and maximum genotype values. Previous studies also showed the same high phenotypic diversity among genotypes (Yousaf et al., 2021, 2024).

### 4.1 The extent of double reduction

According to Nemorin et al. (2012), autotetraploidy is characterized by the occurrence of double reduction events, which results in decreased heterozygosity. It is a genetic process involving the recombination of homologous chromosomes during meiosis but is frequently neglected in tetraploid crops. We investigated the rate of DRP in both M1 and M2 by considering different assumptions of double reduction rates from low to high. The estimated amount of DRP corresponds to the maximum of the marginal likelihood computed under different assumptions on the rate of DRP. Higher log-likelihood values indicate a better fit of the model to the data. DRP at 0.05 or lower had the smallest -2*log(L), indicating the best model fit when the rate of double reduction was very low. Slater et al. (2014) revealed similar findings, demonstrating that different proportions of double reduction (how often this genetic event happens) affect the traits observed in the offspring and concluded that the impact of double reduction (average DRP) rate was approximately 0.1. In potatoes, the chance of double reduction ranges from 0 at the centromere and increases towards the telomere (Bourke et al., 2015). In the investigated population, the rate of double reductions was found to be low and is not expected to have a significant influence on the amount of genetic variance in the population.

### 4.2 Variance components

The estimated variance components showed that additive genetic variance and residual variance in all traits were more important than genetic variance due to non-additive genetic effects, interaction [genotype (G) x environment (E)], and SCA. These results align with other authors concerning the preponderance of additive genetic effect for yield-related traits (Cabello et al., 2014; Paget et al., 2014; Tripura et al., 2016). The significant additive genetic variance observed in DMC, along with little to no non-additive genetic variance, indicates that DMC is mostly affected by additive genetic effects. Ruiz de Arcaute et al. (2022) revealed that GCA had significant effects on variables such as specific gravity (proxy of dry matter content), emphasizing the importance of additive genetic effects in passing these traits to offspring. The RY, GR, and WNG traits showed substantial additive genetic variance, but non-additive genetic variance was also modest when compared to the additive genetic variance, indicating that non-additive effects have a minimal impact. This shows that line differences are primarily due to additive genetic effects and less due to interactions between genes or between parental combinations. Mishra et al. (2017) observed low genetic gain for characteristics related to yield, such as the number of tubers per plant and plant emergence percentage (germination), and suggested that non-additive gene effects exist. Similarly, Thompson and Mendoza (1984) estimated genetic variance for 11 potato traits and discovered non-additive genetic variance in the yield-related traits. The other non-additive effects were calculated as SCA. The estimated variance component associated with the SCA effect reflects the contribution of specific parent cross effects to the trait variability. It provides information about the extent to which genetic interaction between specific genotypes impacts the observed trait values. In the current study, all traits showed much higher GCA variance than SCA variance, demonstrating the importance of additive genetic effects in determining the inheritance of these traits. Thus, additive gene actions were much more important than non-additive gene actions (dominance or epistatic interactions) in the expression of these features. This implies that the traits investigated could be improved efficiently through a well-designed selection approach. Tai (1976) also concluded that GCA effects were significant for average weight per tuber, average weight per marketable tuber, and specific gravity.

The genotype x environment interaction explained a sizable fraction of the total phenotypic variance (from 7.3% to 15.7% depending on the trait), with the highest G x E variance found in GR. The significant influence of G x E interaction indicates that the performance of individual genotypes does change with environmental conditions, but this effect is much smaller than the general additive genetic variance. The relatively limited amount of G x E variance might be because the test sites were all within the mainland of Denmark and, therefore, had somewhat similar climatic conditions. This highlights the importance of considering the interaction between genotype and environment when determining an individual’s genetic potential and selecting superior genotypes to ensure that selected genotypes perform well over a range of relevant environments.

The considerable residual variance compared to other variance components in all traits except DMC suggests that the traits are influenced by unexplained environmental sources of variation. Therefore, selection should be based on replicated experiments.

In open-field variety trials, multiple causes of variation might exist within a location, such as variations in nutrient distribution (Haefele and Wopereis, 2005; Allaire et al., 2014), soil particle size (Santra et al., 2008), and soil organisms (Lupatini et al., 2017), all of which contribute to soil heterogeneity. This field variation can lead to even more unexplained variation because of interactions between the soil environment and genotype (Portman and Ketata, 1997). Inter-plot interference may also be a cause of plot-level effects (Kempton, 1997). Inter-plot interference occurs when adjoining plots compete for resources (above or below ground). In potato, cultivars compete for plant height, tuber yield, and dry matter content across plots (Bradshaw, 1994; Connolly et al., 1993). An important means to reduce errors due to residual environmental effects is the use of replicated plots of each variety in randomized testing designs. It can also be reduced by using larger plots, planting similar types close together, and utilizing border plants surrounding each plot (Bradshaw, 1994, 2021; Kempton, 1997). Additive genetic variance was the most important factor impacting the traits, accounting for most of the observed variation. Although non-additive genetic effects and residuals exist, their impact is relatively low and may be efficiently handled by replicating plots across trials and years. This implies that while environmental factors such as temperature, precipitation, soil quality, and disease pressure interact with genetic factors, the additive genetic component is a fundamental driver of trait variation.

### 4.3 Broad- and narrow-sense heritability

The DMC trait showed a very high heritability, indicating that selective breeding based on individual phenotypic performance is likely to result in significant genetic improvement. Seid et al. (2020) and Adams et al. (2023) found high heritability of DMC, which is in line with our results. More modest heritability was found in RY, GR, and WNG, indicating that both additive and non-additive genetic effects contribute to the total phenotypic variation. Breeding techniques should account for both additive and non-additive genetic effects in the prediction applied. Cabello et al. (2014) reported low to modest heritability for tuber yield traits within the Andigenum group of potatoes, similar to our results. Furthermore, Sood et al. (2020) and Slater et al. (2014) observed low to moderate heritability related to tuber yield traits and moderate heritability for senescence-related traits.

### 4.4 Accuracy ratio and prediction ability

In breeding programs, using the most effective models for predicting genetic values is critical for enhancing crop potential. Model comparison and accuracy assessment are critical steps in this process. Breeders can make informed decisions that are consistent with the underlying hypothesis of their breeding aims by comparing the PA of various models. Commonly used methods for evaluating models and assessing prediction accuracy include CV and the log-likelihood ratio test (Allen, 1974; Stone, 1974; Geisser, 1975; Hastie et al., 2001). In our study, we used two CV strategies (5-Fold and LBCO). Previous research has revealed that a *k*-fold CV is an effective strategy for model selection (Ron, 1995; Ortiz et al., 2023). In contrast, the LBCO better represents the conditions in a breeding scenario in which new generation/lines must be predicted before phenotypes can be obtained. Our results revealed that RC was significantly improved when SCA was included in the model. This means that obtaining the phenotype significantly increases prediction accuracy. In cases when SCA effects are significant, they can account for a portion of the variance previously attributed to additive genetic effects.

Using random 5-Fold cross-validation may result in close relatives in both the training and validation sets, leading to high PA. In contrast, LBCO CV ensures temporal consistency by selecting particular time periods for training and testing, more closely mimicking the real-world scenario (Arlot and Celisse, 2010; Zhang and Yang, 2015). We noted a high PA for the DMC trait in both CVs, while we observed moderate PA for the RY, GR, and WNG traits under 5-Fold and LBCO cross-validation. The 5-fold CV gave a high PA as compared to LBCO, which is because all full and half siblings are masked in LBCO but not in 5-Fold CV. Furthermore, GR exhibited a lower PA in CV2, and PA was moderate in CV1. GR is a complex trait highly impacted by environmental effects. We found low heritability and large variation among lines that were attributed to environmental factors. Although the GR predictions were good for the selection of genotypes based on germination, further increasing the training population size of breeding lines could improve predictions specifically for yield and enhance breeding value estimation for yield improvement (Sood et al., 2020).

The prediction accuracy is also affected by the relationships between individuals in the training population and individuals in the validation population, which is why accuracy in the 5-Fold CV is larger than prediction accuracy in the LBCO CV procedure.

In potato, out of 4,397 cultivars cultivated worldwide, 14.9% have been bred from 15 genotypes (Li et al., 2018), which indicates that common parents have been used in potato breeding across the globe. Therefore, increasing the depth of the pedigree might improve the description of additive genetic relationships in the matrix and better account for all relationships in the population under study. Including genomic information from dense markers will also contribute to a better description of cryptic relationships between individuals due to the historical use of common parents.

The model that included the effects of SCA had a much better fit to the data. SCA reflects the genetic variation that arises from dominance and epistatic interactions between alleles inherited from the parents involved in a mating. These interactions lead to deviations from purely additive genetic expectations, highlighting the importance of non-additive gene action in trait expressions. By adding SCA effects into evaluation models, breeders can exploit genetic interactions between parents to discover superior clones for the market, but for the selection of parents, only the additive effects should be considered because non-additive effects are lost in future generations due to recombination.

Pedigree-based EBVs could be an effective breeding strategy until genomic selection can be routinely implemented. Decisions about parent selection based on EBVs and GEBVs will ultimately enable new, improved high DMC, yielding potato varieties with a range of senescence timings, from early to late.

## 5 Conclusion

This study focused on estimating the rate of double reductions, which is crucial for refining genetic models and improving the accuracy of breeding value predictions and a source of variance affecting traits such as DMC, RY, GR, and WNG. The aim was to quantify the importance of GCA and SCA, enabling breeders to make informed decisions when selecting parental combinations to optimize genetic gain. At the same time, accounting for the influence of environmental factors on trait variability helps breeders identify stable genotypes that perform well across different conditions. Our investigation estimated the occurrence of double reductions and observed that a model with a DRP rate of 0.05 or lower provided the best fit to the data so that the effects of DRP on the population studied were low.

Considerable additive genetic variance and heritability (*h*²) were found for DMC, indicating great potential for improvement through selective breeding, whereas heritability for RY, GR, and WNG were more moderate. However, non-additive genetic variance was low for RY, GR, and WNG, indicating that non-additive influences had a limited impact on these traits. These findings emphasize the importance of additive genetic variance in determining trait heritability, especially for DMC. Additionally, our findings highlighted the need to include SCA in genetic models, as SCA played a substantial role in capturing trait variability. Model comparison using cross-validation and the log-likelihood ratio test revealed that including SCA improved the model’s accuracy in capturing trait variability. These comparisons highlight the need for comprehensive genetic models that account for both additive and non-additive genetic effects and G x E interactions, improving the predictive value of breeding methods for important traits in tetraploid potatoes.

## Data Availability

The original contributions presented in the study are included in the article/**Supplementary Material**. Further inquiries can be directed to the corresponding author.
