# Supplementary material for: Rate of double reduction and genetic variability in yield, quality, and senescence related traits in tetraploid potato (Solanum tuberosum L.)
Source: Front Plant Sci. 2025 Apr 4;16:1560123. doi: 10.3389/fpls.2025.1560123 (PMC12007407; doi:10.3389/fpls.2025.1560123)
Supplement: Supplementary file 1 [file DataSheet1.pdf]

Table S1 Phenotyping descriptions of potato varieties investigated during trials (2003-2021)

**Trait**	**Phenotype description**
Dry Matter Content (DMC)	2500 to 3000 gm potatoes were taken and weighed above and under water by using "hydrometer". The dry matter was automatically calculated by the weighing system by using the following empirical equation: $$\text{Dry matter content (DMC)} = 214\left(\frac{\text{OWW}}{\text{OWW}-\text{UWW}}\right) - 0.988$$ Where OWW is over-water weight, while UWW is under water weight. The above formula is ideal with 3-4 kg sample size but found to be quite robust even for smaller samples size [24].
Relative Yield (RY)	General yield (GY) is calculated in Hkg from yield harvested from each plot using the following formula: $$\text{General yield (GY)} = \frac{\left(\frac{\text{Yield per plot}}{\text{Number of plants per plot (NOP)}} \times 40000\right)}{100}$$ Where 40000 is the number of tubers required for planting at one-hectare field, whereas NOP is number of plants calculated per plots. Further divided with 100 as per equation above to obtained hectokilograms yield (reference from Danespo protocol). Further the relative yield (RY) of each data/entry was calculated in percentage from general yield (GY) as described below: $$\text{Relative yield (RY)} = \frac{\text{GY per plot}}{\text{GY}_{\text{mean}}} \times 100$$ Where the mean values of GY (GY_{mean}) are measured for the entire trial and general yield is relative to the trial varieties.
Germination (GR)	It was measured according to the Danespo potato descriptors. As per descriptor, germination was classified from 1 to 9 different classes i.e.,<br>1: the last to emerge in the field, 9: the very first to emerge in the field
Withering (WNG)	Withering was also measured according to the Danespo potato descriptors. As per descriptor, WNG was also classified from 1 to 9 different classes i.e.,<br>1. No sign of withering, 9: Plant completely gone.

Table S2. Completeness of pedigree by year (a) and market segment (b) was presented below

miss Par (missing parent)

a

**Year**	**Individuals**	**miss par1**	**miss par1(%)**	**miss-par2**	**miss-par2(%)**	**Both par miss**	**both par miss(%)**	**both par known**	**both par known(%)**	**par and grand par known**	**par and grand par known(%)**	**max # of generations known**	**Average**
2003	908	19	2.0	19	2.0	19	2.0	289	31.8	600	66.0	4.3	2.22
2004	656	29	4.4	29	4.4	29	4.4	199	30.3	428	65.2	4.3	2.2
2005	604	10	1.6	10	1.6	10	1.6	211	34.9	383	63.4	3.3	2.22
2006	551	12	2.1	12	2.1	12	2.1	213	38.6	326	59.1	4.1	2.23
2007	667	8	1.1	8	1.1	8	1.1	234	35.0	425	63.7	4.1	2.23
2008	714	7	0.9	7	0.9	7	0.9	211	29.5	496	69.4	3.9	2.25
2009	573	7	1.2	8	1.3	7	1.2	174	30.3	392	68.4	3.9	2.35
2010	658	17	2.5	18	2.7	17	2.5	202	30.6	464	70.5	4.3	2.37
2011	1089	15	1.3	18	1.6	15	1.3	219	20.1	854	78.4	4.4	2.59
2012	1145	6	0.5	7	0.6	6	0.5	238	20.7	901	78.6	4.6	2.58
2013	889	4	0.4	4	0.4	4	0.4	185	20.8	700	78.7	4.5	2.67
2014	1228	8	0.6	8	0.6	8	0.6	351	28.5	869	70.7	4.5	2.53
2015	1446	33	2.2	33	2.2	32	2.2	394	27.2	1018	70.4	4.5	2.48
2016	1426	20	1.4	20	1.4	19	1.3	395	27.6	1010	70.8	4.5	2.51
2017	1659	24	1.4	24	1.4	23	1.3	496	29.8	1138	68.5	4.4	2.44
2018	1856	16	0.8	17	0.9	15	0.8	532	28.6	1308	70.4	4.5	2.48
2019	1444	26	1.8	26	1.8	26	1.8	364	25.2	1054	72.9	4.5	2.4
2020	3117	10	0.3	10	0.3	10	0.3	455	14.5	2652	85.0	4.9	2.81
2021	1109	13	1.1	12	1.0	12	1.0	226	20.3	870	78.4	4.5	2.6

b

Year	Individuals	miss par1	miss par1(%)	miss-par2	miss-par2(%)	both par miss	both par miss(%)	both par known	both par known(%)	par and grand par known	par and grand par known(%)	max # of generations known	Average
Crisps (C)	2127	11	0.51	11	0.51	11	0.51	218	10.24	1898	89.23	4.8	2.94
Starch (F)	4570	33	0.72	34	0.74	33	0.72	664	14.52	3873	84.74	4.9	2.76
Table (S)	7092	104	1.46	109	1.53	103	1.45	2288	32.2	4698	66.24	4.5	2.32

Table S3. REML estimates of variance component and heritabilities for the traits of potato under studied with baseline and SCA models.

Average_Diagonal	Average_Diagonal	D.R*	Genetic		Interaction (Id*Y*T)	SCA	residual	*h2*	H2
			**$\sigma^2 a$	***$\sigma^2 na$					
DM_M1	1.065	0.05	3.1	0.25	0.36	-	0.85	0.69	0.75
DM_M2			3.2	0.01	0.36	0.27	0.84	0.70	0.82
RY_M1			84.3	34.5	38.3	-	185.9	0.26	0.36
RY_M2			83.3	28	39.2	7.5	186	0.25	0.46
GR_M1			0.34	0.15	0.18	-	0.46	0.31	0.44
GR_M2			0.3	0.13	0.18	0.054	0.46	0.28	0.60
WNG_M1			0.51	0.22	0.18	-	0.41	0.4	0.56
WNG_M2			0.48	0.19	0.18	0.056	0.41	0.38	0.69

*Double reduction
** Additive genetic effect
*** Non-additive genetic effect

Table S4. Accuracy of predicting observatiosn and relative accuracy of reduced dataset predicitosn compared with full dataset predictions using5-Fold and Leave-one-breeding cycle out cross validation with baseline and SCA model.

Models	D.R	DMC		RY		GR		WNG	
		*1	**2	1	2	1	2	1	2
5_FOLD_baseline	0.05	0.95	0.812	0.878	0.296	0.839	0.352	0.907	0.531
5_FOLD_SCA		0.949	0.813	0.882	0.3	0.849	0.344	0.913	0.528
LBCO_baseline		0.904	0.726	0.731	0.216	0.599	0.18	0.771	0.409
LBCO_SCA		0.906	0.728	0.741	0.223	0.627	0.18	0.78	0.417

*1 = cor $(\hat{a}_F, \hat{a}_R)$
**2 = cor $(\bar{y}_C, \hat{a}_R)$

The correlation between $\bar{y}_c$ the line average after correction for fixed effects cannot be higher than the square root the heritability of $\bar{y}_c$. This heritability was calculated as;

$$\hat{h}_{\bar{y}_c} = \frac{d(A)\,\hat{\sigma}_a^2}{d(A)\,\hat{\sigma}_a^2 + \hat{\sigma}_g^2 + \frac{\hat{\sigma}_i^2}{\bar{n}_i} + \frac{\hat{\sigma}_s^2}{\bar{n}_s} + \frac{\hat{\sigma}_e^2}{\bar{n}_e}} \qquad (1)$$

Where $\bar{n}_i$ were the average number of environments where a clone was tested, $\bar{n}_s$ were the average number of crosses made from specific parental crosses, and $\bar{n}_e$ were the average number of replicated trials per clone.

Table S5. (a) Heritability estimates for $\bar{y}_c$ (sqrth^2yc) including narrow-sense heritability (h^2) under different genetic models. M1 (without SCA) and M2 (with SCA). (b) Theoretical maximum cor($\bar{y}_c$/sqrth^2yc) of 5-fold and leave breeding cycle out (LBCO).

**(a)**	**DM**	**RY**	**GR**	**WNG**
M1	0.89	0.64	0.67	0.72
M2	0.92	0.65	0.66	0.72
**(b)**	**5_fold**			
	**DM**	**RY**	**GR**	**WNG**
M1	0.91	0.46	0.52	0.73
M2	0.88	0.46	0.52	0.73
**LBCO**				
M1	0.81	0.33	0.26	0.56
M2	0.79	0.34	0.27	0.57

Figure S1 Portrays 5-Fold cross validation scheme used in the study.

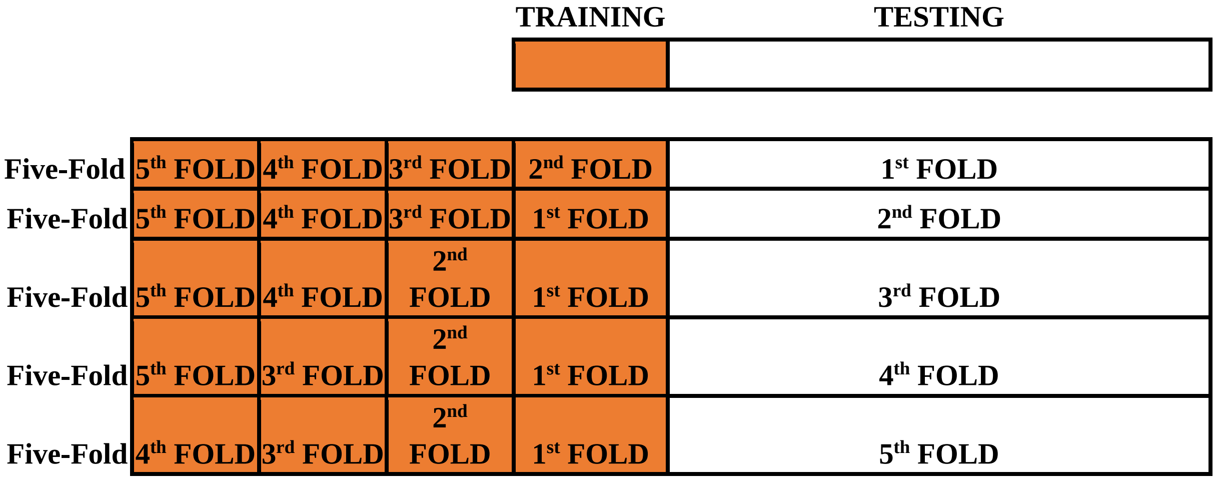

Figure S2 Portrays leave-one-breeding-cycle-out cross validation scheme used in the study.

Training set	Testing set

	Year	Year	Year	Year	Year	Year	Year	So on
**Iteration 1**	Breeding_Year	Breeding_Year	Breeding_Year	Breeding_Year	Breeding_Year	Breeding_Year	Breeding_Year	Breeding_Year
**Iteration 2**	Breeding_Year	Breeding_Year	Breeding_Year	Breeding_Year	Breeding_Year	Breeding_Year	Breeding_Year	Breeding_Year
**Iteration 3**	Breeding_Year	Breeding_Year	Breeding_Year	Breeding_Year	Breeding_Year	Breeding_Year	Breeding_Year	Breeding_Year
**Iteration 4**	Breeding_Year	Breeding_Year	Breeding_Year	Breeding_Year	Breeding_Year	Breeding_Year	Breeding_Year	Breeding_Year
**Iteration 5**	Breeding_Year	Breeding_Year	Breeding_Year	Breeding_Year	Breeding_Year	Breeding_Year	Breeding_Year	Breeding_Year
**Iteration 6**	Breeding_Year	Breeding_Year	Breeding_Year	Breeding_Year	Breeding_Year	Breeding_Year	Breeding_Year	Breeding_Year
**Iteration 7**	Breeding_Year	Breeding_Year	Breeding_Year	Breeding_Year	Breeding_Year	Breeding_Year	Breeding_Year	Breeding_Year
**So on**	Breeding_Year	Breeding_Year	Breeding_Year	Breeding_Year	Breeding_Year	Breeding_Year	Breeding_Year	Breeding_Year
